# Cortical microstructure in young onset Alzheimer's disease using neurite orientation dispersion and density imaging

**DOI:** 10.1002/hbm.24056

**Published:** 2018-03-25

**Authors:** Thomas D. Parker, Catherine F. Slattery, Jiaying Zhang, Jennifer M. Nicholas, Ross W. Paterson, Alexander J.M. Foulkes, Ian B. Malone, David L. Thomas, Marc Modat, David M. Cash, Sebastian J. Crutch, Daniel C. Alexander, Sebastien Ourselin, Nick C. Fox, Hui Zhang, Jonathan M. Schott

**Affiliations:** ^1^ Department of Neurodegenerative Disease Institute of Neurology, UCL London United Kingdom; ^2^ Department of Computer Science and Centre for Medical Image Computing UCL London United Kingdom; ^3^ Department of Medical Statistics, London School of Hygiene and Tropical Medicine London United Kingdom; ^4^ Neuroradiological Academic Unit, Department of Brain Repair and Rehabilitation UCL Institute of Neurology London United Kingdom; ^5^ Leonard Wolfson Experimental Neurology Centre UCL Institute of Neurology London United Kingdom; ^6^ Translational Imaging Group, Centre for Medical Image Computing UCL London United Kingdom

**Keywords:** Alzheimer's disease, dementia, diffusion MRI, grey matter, neurite orientation dispersion and density imaging, neurodegenerative disorders

## Abstract

Alzheimer's disease (AD) is associated with extensive alterations in grey matter microstructure, but our ability to quantify this in vivo is limited. Neurite orientation dispersion and density imaging (NODDI) is a multi‐shell diffusion MRI technique that estimates neuritic microstructure in the form of orientation dispersion and neurite density indices (ODI/NDI). Mean values for cortical thickness, ODI, and NDI were extracted from predefined regions of interest in the cortical grey matter of 38 patients with young onset AD and 22 healthy controls. Five cortical regions associated with early atrophy in AD (entorhinal cortex, inferior temporal gyrus, middle temporal gyrus, fusiform gyrus, and precuneus) and one region relatively spared from atrophy in AD (precentral gyrus) were investigated. ODI, NDI, and cortical thickness values were compared between controls and patients for each region, and their associations with MMSE score were assessed. NDI values of all regions were significantly lower in patients. Cortical thickness measurements were significantly lower in patients in regions associated with early atrophy in AD, but not in the precentral gyrus. Decreased ODI was evident in patients in the inferior and middle temporal gyri, fusiform gyrus, and precuneus. The majority of AD‐related decreases in cortical ODI and NDI persisted following adjustment for cortical thickness, as well as each other. There was evidence in the patient group that cortical NDI was associated with MMSE performance. These data suggest distinct differences in cortical NDI and ODI occur in AD and these metrics provide pathologically relevant information beyond that of cortical thinning.

## 1 INTRODUCTION

There is considerable interest in quantifying the neuropathological alterations associated with Alzheimer's disease (AD) using in vivo imaging techniques. An extensive body of literature utilizing volumetric T1‐weighted MRI has highlighted that widespread cortical atrophy occurs in multiple brain regions affected by AD pathology (Apostolova et al., 2007; Becker et al., 2011; Bourgeat et al., 2010; Chetelat et al., 2012; Dickerson et al., 2009, 2001; Fox et al., 2001; Frisoni, Prestia, Rasser, Bonetti, & Thompson, 2009; Harper et al., 2017; Jack et al., 2015; LaPoint et al., 2017; Lerch et al., 2004; Prestia et al., 2010; Singh et al., 2006; Thompson et al., 2003; Whitwell et al., 2008, 2013; Xia et al., 2017). Decreased dendritic arborization, loss of dendritic spines, and loss of synapses have been observed in neuropathological studies of AD and have been shown to be associated with cognitive deficits (Baloyannis, Manolides, & Manolides, 2011; Davies, Mann, Sumpter, & Yates, 1987; DeKosky and Scheff, 1990; Masliah, Terry, DeTeresa, & Hansen, 1989; Scheff, Price, Schmitt, & Mufson, 2006; Scheff, Price, Schmitt, DeKosky, & Mufson, 2007; Scheff, Price, Schmitt, Scheff, & Mufson, 2011; Scheff and Price, 1993; Terry et al., 1991).

However, research into imaging techniques that can quantify AD‐related differences in cortical microstructure in vivo has been relatively limited. Diffusion MRI, which measures the directionally‐dependent diffusion of water molecules, has been extensively applied to the study of white matter in AD, and has also been proposed as a method of assessing the microstructural properties of grey matter (Weston, Simpson, Ryan, Ourselin, & Fox, 2015). Many diffusion MRI studies investigating AD published thus far have used diffusion tensor imaging (DTI) analysis. Differences in DTI metrics in the cortex have been associated with clinically established AD (Rose, Janke PhD, & Chalk, 2008), conversion to AD from mild cognitive impairment (Scola et al., 2010), cognitive decline (Jacobs et al., 2013), cerebrospinal fluid (CSF) biomarkers of AD pathology in cognitively normal individuals (Montal et al., 2017), and have been used to differentiate dementia subtypes (Kantarci et al., 2010).

A limitation of the DTI model is that it can only assess diffusion of water molecules across the entire voxel. An important development has been multi‐shell diffusion MRI techniques that allow more advanced modeling of the diffusion signal, such as neurite orientation dispersion and density imaging (NODDI) (Zhang, Schneider, Wheeler‐Kingshott, & Alexander, 2012); a clinically feasible approach that can derive tissue‐specific microstructural information from multiple compartments within a voxel. The NODDI model assumes that water molecules in neuronal tissue can be considered within three separate compartments: (a) free water, representing CSF; (b) restricted water, representing neurites; and (c) hindered water, representing diffusion within glial cells, neuronal cell bodies and the extracellular environment. This enables estimation of the neurite density index (NDI), with values ranging from 0 to 1 (higher values reflecting increased neurite density); and orientation dispersion index (ODI), which provides information regarding the extent of neurite dispersion (values of 0 equating to no dispersion and values of 1 equating to full dispersion). This and related modelling approaches in the context of grey matter provides information regarding the structural organization of neuronal dendritic trees. Furthermore, the capacity to model the fraction of free water reduces the risk of partial volume effects from CSF contamination influencing NODDI metrics, something which is not explicitly considered within the DTI model (Zhang et al., 2012) and often significantly influence DTI based studies of grey matter in AD (Henf, Grothe, Brueggan, Teipel, & Dyrba, 2017; Jeon et al., 2012). Differences in cortical NODDI metrics have been reported in ageing (Nazeri et al., 2015), Parkinson's disease (Kamagata et al., 2017), schizophrenia (Nazeri et al., 2016), multiple sclerosis (Granberg et al., 2017), and transgenic murine models of AD tauopathy (Colgan et al., 2016). To date, no studies have been published assessing cortical microstructure using NODDI in human AD.

Within the wide clinical spectrum of AD, an interesting and relatively understudied patient group is those with young onset AD (YOAD—defined as symptom onset < 65 years). Although less prevalent than in older age ranges, AD still represents the biggest cause of dementia before the age of 65 (Harvey, Skelton‐Robinson, & Rossor, 2003) and often poses a significant diagnostic challenge and is more likely to present with nonamnestic phenotypes (Rossor, Fox, Mummery, Schott, & Warren, 2010; Slattery et al., 2017). An advantage of studying such patients is they often have fewer age‐related co‐morbidities than patients with later onset disease, where co‐existing pathologies can confound analyses of brain structure. Studies specifically focusing on grey matter atrophy in YOAD have revealed extensive cortical atrophy compared to healthy controls (Cho et al., 2013; Frisoni et al., 2007; Harper et al., 2017; Ishii et al., 2005; Möller et al., 2013; Ossenkoppele et al., 2015). Similarly, recent data from tau PET imaging studies have provided evidence of diffuse neurofibrillary tangle deposition in the neocortex in YOAD (Ossenkoppele et al., 2016).

In this article, we applied a cross‐sectional surface‐based region of interest (ROI) cortical analysis approach using NODDI metrics and cortical thickness derived from T1‐weighted images to a population of patients with YOAD, as well as age‐matched healthy controls to investigate the following hypotheses: (a) differences in NODDI metrics mirror patterns of macroscopic atrophy in regions known to undergo early AD‐related pathological change, (b) NODDI metrics provide distinct information regarding AD‐related pathology above and beyond measures of cortical thickness, (c) differences in NODDI metrics are evident in a brain region vulnerable to AD pathology but known to be relatively spared of atrophy (the precentral gyrus) (Albers et al., 2015; Braak and Braak, 1991; Buchman and Bennett, 2011; Frisoni et al., 2007; Horoupian and Wasserstein, 1999; Suvà et al., 1999), implying NODDI is a more sensitive marker of cortical pathology than macroscopic measures, and (d) NODDI metrics are associated with a global score of cognition.

## 2 MATERIALS AND METHODS

### 2.1 Participants

A total of 45 patients meeting consensus criteria for probable AD (McKhann et al., 2011) with symptom onset <65 years were recruited prospectively from 2013 to 2015 from a specialist cognitive disorders clinic (Slattery et al., 2017). All participants underwent detailed clinical assessment, which included the Mini–Mental State Examination(MMSE) (Folstein, Folstein, & McHugh, 1975). Documentation of the age at symptom onset and the presenting cognitive symptom were recorded for all patients. Patients were classified as having a typical (McKhann et al., 2011) or atypical AD (Tang‐Wai et al., 2004) phenotype according to published criteria. Twenty‐eight of those patients were classified as having typical AD (amnestic presentation), while 17 patients were classified as having atypical AD (14 of which had a visual symptom‐led posterior cortical atrophy phenotype). Twenty‐four participants with no history of cognitive concerns were recruited as healthy controls matched for mean age and gender and were predominantly spouses of the YOAD patients. Ethical approval was obtained from the National Hospital for Neurology and Neurosurgery Research Ethics Committee and written informed consent was obtained from all the participants.

### 2.2 MRI acquisition

All participants were scanned on the same Siemens Magnetom Trio (Siemens, Erlangen, Germany) 3 Tesla MRI scanner using a 32‐channel phased array receiver head coil. Sequences utilized for this analysis included sagittal 3D MPRAGE T1‐weighted volumetric MRI (TE/TI/TR = 2.9/900/2,200 ms, matrix size 256 × 256 × 208, voxel size 1.1 × 1.1 × 1.1 mm^3^), *B*_0_ field mapping (TE1,2/TR = 4.92,7.38/688 ms, matrix size 64 × 64 × 55, voxel size 3 × 3 × 3 mm^3^; total time = 9 min 23 s), and a three shell diffusion sequence optimized for NODDI processing(Slattery et al., 2017) (64, 32, and 8 diffusion‐weighted directions at *b* = 2,000, 700, and 300 s/mm^2^; 14 interleaved *b* = 0 images; 55 slices; voxel size 2.5 × 2.5 × 2.5 mm; TE/TR = 92/7,000 ms; total time = 16 min 13 s). A twice‐refocused spin echo was utilized to minimize distortion effects from eddy‐currents. All scans were visually assessed for quality control purposes, based on coverage and movement artefact.

### 2.3 Cortical reconstruction of structural imaging

**Figure 1:**
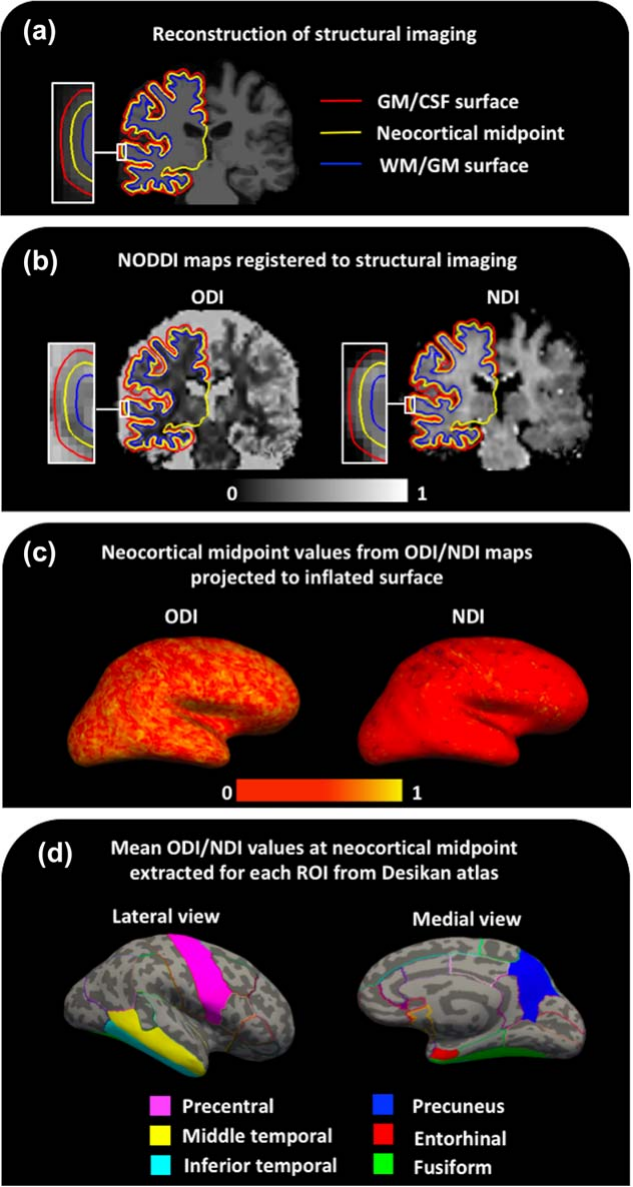
Steps involved in surface‐based region of interest cortical analysis of NODDI maps. (a) Reconstruction of structural imaging, (b) NODDI maps registered to structural imaging, (c) neocortical midpoint values from ODI/NDI maps projected to inflated surface, and (d) mean ODI/NDI values at each neocortical midpoint extracted for each ROI from Desikan atlas. Key: GM = grey matter; WM = white matter; CSF = cerebrospinal fluid; ODI = orientation dispersion index; NDI = neurite density index; ROI = region of interest [Color figure can be viewed at http://wileyonlinelibrary.com]

Automated surface‐based reconstruction of T1‐weighted volumetric images was performed using Freesurfer version 6.0 (http://surfer.nmr.mgh.harvard.edu/). This procedure has been described in detail and validated in previous publications (Dale, Fischl, & Sereno, 1999; Fischl and Dale, 2000). Briefly, the image processing involves intensity normalization, skull stripping, segmentation of white matter, and tessellation of the grey/white matter boundary. This surface is then used as the starting point for a deformable surface algorithm to find the grey matter/white matter and grey matter/cerebrospinal fluid surfaces. All cortical segmentations were visually inspected slice by slice for accuracy (Figure 1, panel a).

### 2.4 Cortical thickness estimation

Cortical thickness estimates were obtained using Freesurfer by calculating the mean distance between grey matter/white matter and grey matter/cerebrospinal fluid surfaces at each vertex across the cortical mantle (Dale et al., 1999; Fischl and Dale, 2000). These thickness measures were then mapped to the inflated surface of each participant's reconstructed brain.

### 2.5 Diffusion‐weighted MRI processing

Images were confirmed by visual inspection to have minimal eddy‐current distortion and were corrected for motion by rigidly registering each diffusion‐weighted image to the first *b* = 0 image from the *b* = 2,000 s/mm^2^ series using FLIRT (Jenkinson, Bannister, Brady, & Smith, 2002; Jenkinson and Smith, 2001). ODI and NDI maps were estimated using the NODDI Matlab toolbox (http://www.nitrc.org/projects/noddi_toolbox). The acquired field maps were then registered to the first *b* = 0 and used to correct the ODI and NDI maps for EPI distortion (Daga et al., 2014). To provide a mask for field map processing a total intracranial mask (Malone et al., 2015) generated from SPM12 (http://www.fil.ion.ucl.ac.uk/spm) segmentation was propagated from the T1 to diffusion volumes by a union of direct affine transformation and nonlinear transformation to provide maximal coverage before and after susceptibility correction. Structural T1 to diffusion *b* = 0 and diffusion to field map magnitude registration was carried out using Niftyreg (Modat et al., 2014) (Figure 1, panel b). All registrations were visually inspected for accuracy. ODI and NDI values were then sampled from the midpoint of the cortical ribbon (i.e. 50% of the cortical thickness along the surface normal to the grey matter/white matter surface) and subsequently projected to an inflated surface to create ODI and NDI surface maps using the mri_vol2surf function in Freesurfer (Figure 1, panel c).

### 2.6 Cortical ROIs

Mean cortical thickness, ODI, and NDI values across both hemispheres were extracted for a number of a priori cortical ROIs from Freesurfer's gyral based Desikan parcellation using the *mris_anatomical_stats* function in Freesurfer (see panel d in Figure 1). Five of the ROIs were chosen on the basis of being early sites of neuropathological change in AD. These included four regions that comprise the Mayo Clinic cortical signature of AD (entorhinal cortex, inferior temporal gyrus, middle temporal gyrus, and fusiform gyrus) (Jack et al., 2015; Schwarz et al., 2016), all of which have been implicated to undergo grey matter atrophy at a relatively early stage of AD (Braak and Braak, 1991; Dickerson et al., 2011; Jack et al., 2017; Petersen et al., 2016; Weston et al., 2016) and the precuneus, which has been reported to undergo more pronounced atrophy in YOAD patients (Ishii et al., 2005; Karas et al., 2007; Möller et al., 2013). To investigate if cortical NODDI metrics are potentially a more sensitive marker of AD pathology than cortical macrostructural metrics, the precentral gyrus (primary motor cortex) was included as a ROI. The precentral gyrus is relatively spared from atrophy in YOAD (Frisoni et al., 2007), but there is evidence from neuropathological studies that the primary motor cortex is vulnerable to significant levels of AD related pathology (Albers et al., 2015; Braak and Braak, 1991; Buchman and Bennett, 2011; Horoupian and Wasserstein, 1999; Suvà et al., 1999).

### 2.7 Exclusions

One patient with typical AD was found to have an autosomal dominant genetic cause of dementia (Beck et al. 2014) and was excluded from the analysis. YOAD patients with language‐led or behavioral phenotypes were not included in the analysis due to limited numbers (*n* = 2 and *n* = 1, respectively). One YOAD patient failed Freesurfer processing. Three YOAD patients and two healthy control participants were excluded on the basis of severe motion artefact identified during visual inspection of the diffusion weighted MRI images.

### 2.8 Statistical analysis

Sixty participants (38 YOAD and 22 controls) were included and Stata version 14 was used for analysis. Demographics and clinical characteristics were compared between each group. For continuous characteristics two‐sample *t* tests were used, or where there was a material departure from a normal distribution, a Wilcoxon rank sum test was used. Categorical characteristics were compared between groups using Fisher's exact test due to the relatively small numbers in each group. Unadjusted comparisons of cortical thickness, ODI and NDI between patient groups for each ROI was performed using two‐sample *t* tests, with Satterthwaite's approximation to allow for unequal variances. Given the large voxel size of diffusion‐weighted MRI images and the consequent increased risk of contamination of the cortical ribbon with non‐grey‐matter voxels with cortical thinning, linear regression comparing mean ODI and NDI values between groups after adjustment for mean cortical thickness values in each ROI (with robust standard errors to allow for heteroscedasticity) was also performed. The NODDI model, by construction, treats NDI and ODI as two independent measures that quantify distinct, but complementary aspects of microstructure. To investigate whether YOAD‐related differences in ODI or NDI occurred independent of each other we performed linear regression models in each ROI (again with robust standard errors to allow for heteroscedasticity) where NDI and ODI were corrected for each other, as well as cortical thickness. The association between cortical thickness, ODI, and NDI values for each ROI with MMSE score was assessed using Spearman's correlation coefficient in the YOAD group alone. To preserve a family‐wise error rate of 5% to detect differences in cortical thickness, ODI, and NDI, a threshold of *p* < .008 for formal statistical significance was used after Bonferroni correction for comparison across the 6 ROIs.

## 3 RESULTS

### 3.1 Demographics and clinical characteristics

Basic demographic and clinical data are summarized in Table 1. There were no significant differences in age, gender, or years of education when comparing healthy controls with YOAD patients. As expected, MMSE scores were significantly lower in YOAD patients compared to controls. 27 patients had a typical amnestic presentation and 11 had an atypical visual‐led posterior cortical atrophy presentation. There were no significant differences between typical and atypical AD patients in terms of age, gender, age of symptom onset, disease duration, MMSE score, or years of education (see Supporting Information).

**Table 1:** Demographics, clinical, and genetic characteristics

	HC (*n* = 22)	YOAD (*n* = 38)	*p* value
Age (years)	60.6 (5.6)	61.1 (4.9)	.71^a^
% Female	54.5%	60.5%	.79^b^
Age at onset (years)	n/a	56.3 (4.4)	n/a
Disease duration	n/a	4.8 (2.6)	n/a
MMSE score	29.5 (0.7)	21.2 (4.6)	<.0001^c^
Years of education	16.5 (3.3)	15.5 (2.4)	.15^a^

*Note*. Abbreviations: HC = healthy controls; YOAD = young onset Alzheimer's disease; *n* = number; SD = standard deviation; MMSE = Mini‐Mental State Examination.

All data are mean (SD) unless stated otherwise.

a Two‐tailed *t* test.

b Two‐sided Fisher's exact.

c Wilcoxon rank sum.

### 3.2 Direct comparisons of cortical thickness, ODI, and NDI values in ROIs between patient groups

**Figure 2:**
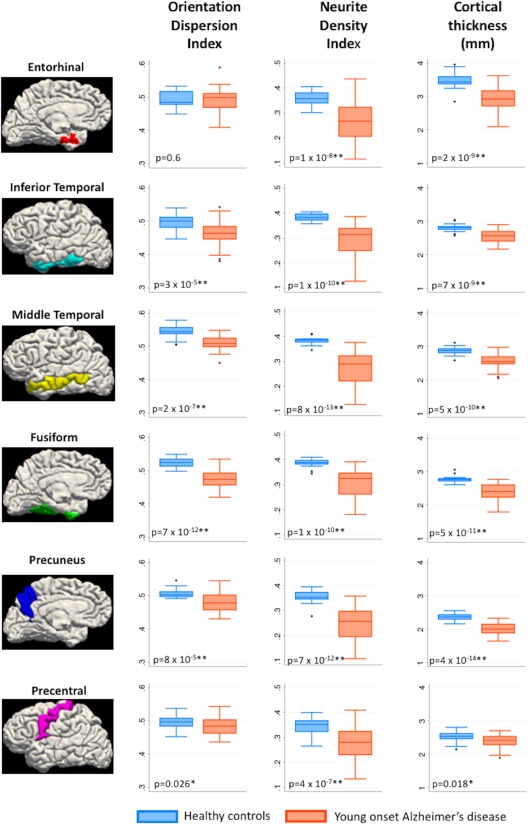
Box plots ‐ cortical thickness, NDI, and ODI in healthy controls and young onset Alzheimer's disease in a priori cortical regions‐of‐interest (NDI = neurite density index; ODI = orientation dispersion index): * *p* < .05 **p < .008. Bonferroni corrected threshold: *p* = .05 divided by 6 (total number of regions of interest) [Color figure can be viewed at http://wileyonlinelibrary.com]

Mean values for cortical thickness, NDI, and ODI for each ROI and their statistical comparisons by participant group are detailed in Table 2 and via boxplots in Figure 2. For all ROIs investigated, NDI was significantly lower in YOAD patients compared to healthy controls (*p* < .008—Bonferroni corrected threshold). Cortical thickness was significantly lower in YOAD patients compared to healthy controls in the ROIs associated with early atrophy in AD (entorhinal, inferior temporal, middle temporal, fusiform, and precuneus) (*p* < .008—Bonferroni corrected threshold), with a trend toward lower cortical thickness in YOAD patients in the precentral ROI (*p* = .01). ODI was lower in YOAD patients compared to healthy controls in the ROIs associated with early atrophy in AD (inferior temporal, middle temporal, fusiform, and precuneus) (*p* < .008—Bonferroni corrected threshold), except the entorhinal cortex where no statistically significant difference was observed. There was also a trend toward lower ODI in YOAD patients in the precentral ROI (*p* = .026). There was no evidence for a difference in NDI or ODI between the typical AD and atypical AD groups, although there was evidence of reduced fusiform cortical thickness in the atypical compared to typical YOAD patients (see Supporting Information).

**Table 2:** Comparisons of cortical thickness, NDI, and ODI for each region of interest by participant group

Region of interest		HC (*n* = 22)	YOAD (*n* = 38)	*p* value
Entorhinal	Cortical thickness (mm)	3.48 (0.24)	2.92 (0.36)	2 × 10^−9^**
	NDI	0.359 (0.028)	0.267 (0.075)	1 × 10^−8^**
	ODI	0.494 (0.022)	0.490 (0.035)	0.6
Inferior temporal	Cortical thickness (mm)	2.81 (0.11)	2.55 (0.19)	7 × 10^−9^**
	NDI	0.383 (0.013)	0.295 (0.062)	1 × 10^−10^**
	ODI	0.499 (0.024)	0.464 (0.035)	3 × 10^−5^**
Middle temporal	Cortical thickness (mm)	2.87 (0.12)	2.55 (0.21)	5 × 10^−10^**
	NDI	0.393 (0.014)	0.273 (0.064)	8 × 10^−13^**
	ODI	0.543 (0.019)	0.511 (0.020)	2 × 10^−7^**
Fusiform	Cortical thickness (mm)	2.76 (0.097)	2.41 (0.24)	5 × 10^−11^**
	NDI	0.389 (0.016)	0.309 (0.057)	1 × 10^−10^**
	ODI	0.525 (0.014)	0.476 (0.029)	7 × 10^−12^**
Precuneus	Cortical thickness (mm)	2.37 (0.10)	2.01 (0.17)	4 × 10^−14^**
	NDI	0.357 (0.025)	0.249 (0.068)	7 × 10^−12^**
	ODI	0.507 (0.013)	0.483 (0.030)	8 × 10^−5^**
Precentral	Cortical thickness (mm)	2.51 (0.16)	2.40 (0.19)	0.018^*^
	NDI	0.346 (0.031)	0.276 (0.064)	4 × 10^−7^**
	ODI	0.498 (0.019)	0.484 (0.026)	0.026^*^

*Note*. Abbreviations: AD = Alzheimer's disease; HC = healthy controls; *n* = number; NDI = neurite density index, ODI = orientation dispersion index; SD = standard deviation.

All data are mean (SD) unless stated otherwise.

*p < .05; **p < .008; Bonferroni corrected threshold: *p* = .05 divided by 6 (total number of regions of interest).

### 3.3 NODDI metrics adjusted for cortical thickness

Differences in mean ODI and NDI values adjusted for mean cortical thickness values in each ROI between healthy controls and YOAD are displayed in Table 3. Following adjustment for cortical thickness, NDI was still significantly lower in YOAD patients compared to controls in the inferior temporal, middle temporal, and precentral ROIs. There was also a trend toward lower NDI in patients in the entorhinal and fusiform regions, with no statistically significant differences observed in the precuneus. Following adjustment for cortical thickness mean ODI for all ROIs except the entorhinal cortex was lower in YOAD patients compared to controls.

**Table 3:** Comparisons of cortical thickness adjusted‐NDI and cortical thickness adjusted‐ODI for each region of interest by participant group

Region of interest		Mean difference HC vs YOAD (*p* value)
Entorhinal	NDI_adjusted for cortical thickness_	−0.048 (0.022^*^)
	ODI_adjusted for cortical thickness_	0.003 (0.73)
Inferior temporal	NDI_adjusted for cortical thickness_	−0.038 (0.002**)
	ODI_adjusted for cortical thickness_	−0.03 (0.006**)
Middle temporal	NDI_adjusted for cortical thickness_	−0.05 (0.0003**)
	ODI_adjusted for cortical thickness_	−0.032 (7 × 10^−6^**)
Fusiform	NDI_adjusted for cortical thickness_	−0.027 (0.011^*^)
	ODI_adjusted for cortical thickness_	−0.03 (0.0003**)
Precuneus	NDI_adjusted for cortical thickness_	−0.026 (0.16)
	ODI_adjusted for cortical thickness_	−0.032 (0.0002**)
Precentral	NDI_adjusted for cortical thickness_	−0.055 (2 × 10^−5^**)
	ODI_adjusted for cortical thickness_	−0.019 (0.004**)

*Note*. Abbreviations: HC = healthy controls; *n* = number; NDI = neurite density index; ODI = orientation dispersion index; SD = standard deviation; YOAD = Young onset Alzheimer's disease.

*p < .05; **p < .008; Bonferroni corrected threshold: *p* = .05 divided by 6 (total number of regions of interest).

### 3.4 ODI and NDI adjusted for each other as well as cortical thickness

Comparisons between YOAD patients and healthy controls of mean NDI adjusted for mean cortical thickness and mean ODI, as well as mean ODI adjusted for mean cortical thickness and mean NDI, for each region of interest are displayed in Table 4. Following adjustment for mean cortical thickness and mean ODI, mean NDI was still significantly lower in YOAD patients compared to controls in the inferior temporal, middle temporal and precentral ROIs. There was also a trend toward lower NDI in patients in the entorhinal, fusiform, and precuneus regions. Following adjustment for cortical thickness and mean NDI, mean ODI for all ROIs except the entorhinal cortex was lower in YOAD patients compared to controls.

**Table 4:** Comparisons between YOAD patients and healthy controls of NDI adjusted for cortical thickness and ODI, as well as ODI adjusted for cortical thickness and NDI, for each region of interest

Region of interest		Mean difference HC vs YOAD (*p* value)
Entorhinal	NDI_adjusted for cortical thickness & ODI_	−0.048 (0.022^*^)
	ODI_adjusted for cortical thickness & NDI_	0.003 (0.8)
Inferior temporal	NDI_adjusted for cortical thickness & ODI_ ODI_adjusted for cortical thickness & NDI_	−0.039 (0.003**) −0.031 (0.007**)
Middle temporal	NDI_adjusted for cortical thickness & ODI_ ODI_adjusted for cortical thickness & NDI_	−0.055 (0.001**) −0.034 (7 × 10^−5^**)
Fusiform	NDI_adjusted for cortical thickness & ODI_	−0.013 (0.27)
	ODI_adjusted for cortical thickness & NDI_	−0.025 (0.001**)
Precuneus	NDI_adjusted for cortical thickness & ODI_	−0.042 (0.022^*^)
	ODI_adjusted for cortical thickness & NDI_	−0.036 (0.0002**)
Precentral	NDI_adjusted for cortical thickness & ODI_	−0.076 (6 × 10^−8^**)
	ODI_adjusted for cortical thickness & NDI_	−0.032 (4 × 10^−6^**)

*Note*. Abbreviations: HC = healthy controls; *n* = number; NDI = neurite density index; ODI = orientation dispersion index; SD = standard deviation; YOAD = Young onset Alzheimer's disease.

*p < .05; **p < .008; Bonferroni corrected threshold: *p* = .05 divided by 6 (total number of regions of interest).

### 3.5 Association with MMSE score

**Figure 3:**
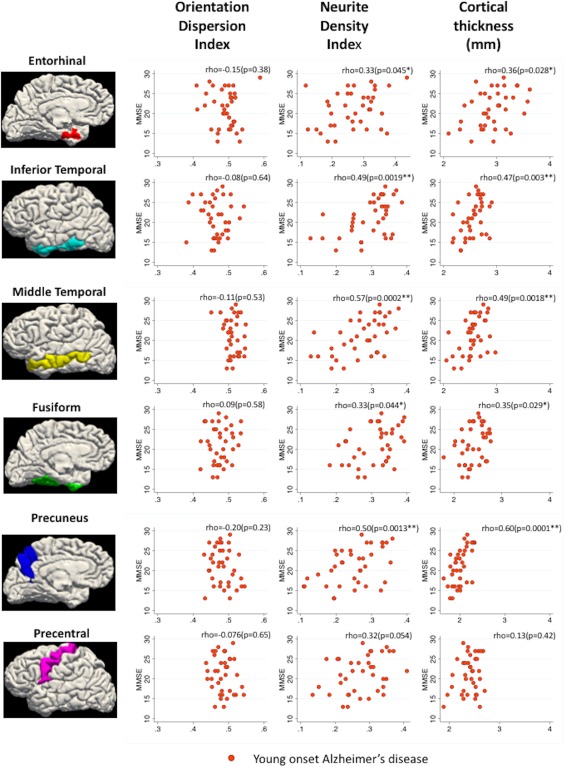
Scatter plots showing relationship between MMSE score (MMSE = mini mental state examination) and orientation dispersion index (ODI)/neurite density index (NDI)/cortical thickness in YOAD patients only: *p < .05, **p < .008. Bonferroni corrected threshold: *p* = .05 divided by 6 (total number of regions of interest) [Color figure can be viewed at http://wileyonlinelibrary.com]

Association of mean NDI, ODI, and cortical thickness for each ROI with MMSE scores in YOAD patients only is displayed in Table 5 and Figure 3. There was evidence that lower MMSE was associated with lower NDI and reduced cortical thickness with the strongest associations in the precuneus, inferior temporal, and middle temporal ROIs. No associations were observed between MMSE and ROI values for ODI. No associations were observed between MMSE and ROI values for cortical thickness, NDI, or ODI in healthy controls (data not shown).

**Table 5:** Association between region of interest metrics (cortical thickness, NDI, and ODI with MMSE)

Region of interest		Association with MMSE score Spearman's rho (*p* value) All YOAD (*n* = 38)
Entorhinal	Cortical thickness (mm)	0.36 (0.028^*^)
	NDI	0.33 (0.045^*^)
	ODI	−0.15 (0.38)
Inferior temporal	Cortical thickness (mm) NDI ODI	0.47 (0.003**) 0.49 (0.0019**) −0.08 (0.64)
Middle temporal	Cortical thickness (mm) NDI ODI	0.49 (0.0018**) 0.57 (0.0002**) −0.11 (0.53)
Fusiform	Cortical thickness (mm)	0.35 (0.029^*^)
	NDI	0.33 (0.044^*^)
	ODI	0.09 (0.58)
Precuneus	Cortical thickness (mm)	0.60 (0.0001**)
	NDI	0.50 (0.0013**)
	ODI	−0.20 (0.23)
Precentral	Cortical thickness (mm)	0.13 (0.42)
	NDI	0.32 (0.054)
	ODI	−0.076 (0.65)

*Note*. Abbreviations: AD = Alzheimer's disease; HC = healthy controls; *n* = number; NDI = neurite density index; ODI = orientation dispersion index; YOAD = Young Onset Alzheimer's disease.

*p < .05; **p < .008; Bonferroni corrected threshold: *p* = .05 divided by 6 (total number of regions of interest).

## 4 DISCUSSION

We report evidence that there are widespread alterations in cortical microstructure in YOAD, with reductions in NDI in YOAD patients compared to healthy controls in all ROIs investigated (entorhinal, middle temporal, inferior temporal, fusiform, precuneus, and precentral) and reductions in ODI in YOAD patients compared to healthy controls in inferior temporal, middle temporal, fusiform, and precuneus ROIs.

In ROIs associated with early atrophy in AD (entorhinal, inferior temporal, middle temporal, fusiform, and precuneus) both mean NODDI metrics (except entorhinal ODI) and cortical thickness differed between healthy controls and those with YOAD. Associations between cortical thickness and diffusion imaging metrics have been reported in brain regions associated with atrophy in AD previously (Jacobs et al., 2013). One potential interpretation of such results is that the decreases in cortical thickness may result in decreases in diffusion metrics because of partial volume effects rather than a purely biological association (Henf et al., 2017; Jeon et al., 2012). This has been a longstanding criticism of diffusion MRI studies focusing on the cerebral cortex in neurodegenerative disease (Weston et al., 2015), given the thin nature of cortex and the limited spatial resolution of diffusion MRI acquisition sequences (2.5 mm × 2.5 mm × 2.5 mm voxel size in this study) leading to increased risk of contamination with non‐grey‐matter voxels. The NODDI model does attempt to account for this by modeling the fraction of free water, with the aim of reducing the risk of partial volume effects from CSF. However, in the context of atrophy, there is still a risk of partial volume effects from adjacent white matter, which is not explicitly accounted for in the NODDI model. To explore the impact of cortical thinning further, mean NDI and ODI values were also adjusted for mean cortical thickness values in each ROI using regression models. After adjustment, the results still demonstrated statistically significant reductions in NDI and ODI when comparing YOAD patients and healthy controls in the majority of the ROIs examined. These results suggest that these microstructural metrics do provide information regarding cortical structure above and beyond cortical atrophy alone. Furthermore, it suggests that differences in NODDI metrics are unlikely to be due to contamination from non‐grey‐matter voxels. Findings in the aforementioned study by Nazeri et al. (2015) describing ODI associations with increasing age also survived adjustment for cortical thickness, providing further evidence that NODDI metrics provide information beyond cortical macrostructure. The difference in precuneus NDI between YOAD patients and controls did not survive adjustment for cortical thickness, although differences in ODI did. The precuneus is one of the earliest brain regions to undergo volume loss in the course of YOAD (Ishii et al., 2005; Karas et al., 2007; Möller et al., 2013) and the patients included in this study all had established disease. This finding is consistent therefore with the hypothesis that whilst neurite loss may be an early feature of the disease, by the time the disease becomes more advanced the likely inherent pathophysiological relationship between NDI and cortical thickness becomes more apparent. As discussed previously, it is however still possible that cortical NDI is more susceptible than ODI to partial volume effects from adjacent subcortical white matter.

We also report evidence that the majority of YOAD‐related differences in ODI and NDI survive correction for each other, as well as cortical thickness. The NODDI model (Zhang et al., 2012) proposes that ODI estimates neurite dispersion, which in the context of grey matter may reflect the degree of complexity of dendritic trees, while NDI is an estimate of neurite density. Although, this requires histopathological confirmation, these data support the notion that ODI and NDI provide estimates of distinct microstructural properties, and they may be differentially affected in YOAD.

It is interesting that there was significantly decreased NDI in the precentral gyrus in YOAD patients compared to healthy controls, while there was only weak evidence that these groups differed on a measure of cortical macrostructure (cortical thickness). This is a region that is relatively spared from atrophy in YOAD (Frisoni et al., 2007), however there is evidence from neuropathological studies that it can be is a site of AD‐related pathology (Albers et al., 2015; Braak and Braak, 1991; Buchman and Bennett, 2011; Horoupian and Wasserstein, 1999; Suvà et al., 1999). Although limited by its cross‐sectional nature these data raise the possibility that such decreases in NDI may precede macroscopically detectable differences in cortical structure and may be a more sensitive marker of AD pathology.

We report evidence that lower NDI values in the precuneus, inferior temporal, and middle temporal were associated with a lower global score of cognition, in the form of MMSE, and may provide information regarding disease severity. The capacity of cortical NODDI to provide information regarding cognitive status is further supported by the fact that associations between cortical differences in NODDI metrics and cognition have also been demonstrated by Nazeri and colleagues in the context of normal ageing (Nazeri et al., 2015) and major psychotic disorders (Nazeri et al., 2016).

Colgan et al have published evidence that murine models of AD tau pathology (rTg4510 transgenic mice) have lower cortical ODI compared to wild type mice but increased cortical NDI. Increased cortical NDI was also associated with an increased percentage of tau immunoreactivity (Colgan et al., 2016). The increases in NDI are discordant with our data, which demonstrated consistent decreases in NDI in AD patients. However, the mean symptom duration of YOAD patients in this study was ∼4.8 years, which according to hypothetical models of pathophysiological change in AD is likely to be decades after initial deposition of hyperphosphorylated tau (Jack et al., 2013, 2010, 2017) making direct comparison to such mouse models difficult. There is evidence from human imaging studies suggesting that apparently paradoxical increases in grey matter volume and cortical thickness may occur in the early stages of AD before a phase of accelerated atrophy (Chételat et al., 2010; Fortea et al., 2010, 2011, 2014; Montal et al., 2017; Pegueroles et al., 2017). It is possible such nonlinear relationships may occur in microstructural properties, but further studies are required to fully elucidate these relationships. In particular, relating measurements of cortical grey matter microstructure with data from in vivo PET radiotracers of beta‐amyloid (Clark et al., 2011; Joshi et al., 2012; Klunk et al., 2004) and tau deposition (Marquié et al., 2015) may be helpful to delineate this relationship more precisely.

YOAD is notable for the degree of phenotypic heterogeneity and our dataset did include a small number of patients (*n* = 11) with an atypical, visual‐led presentation consistent with a clinical diagnosis of posterior cortical atrophy. No clear differences in ODI or NDI were noted when comparing typical and atypical phenotypes in the ROIs chosen for the purpose of this analysis. Previous larger scale analyses have revealed differences in cortical macrostructure (Lehmann et al., 2011) between typical AD and posterior cortical atrophy. In addition to the small sample size, the lack of difference between phenotypes in this analysis may also reflect the fact that the patients included were at a relatively late stage of the disease and that differences in these potentially more sensitive microstructural metrics were well established in both phenotypes at this time point.

Limitations of this study include the fact that five participants were excluded due to diffusion imaging movement artefacts (as well as one participant failing Freesurfer processing). Furthermore, there was a sole focus on AD patients with a young onset and replication in those with later onset AD will be required in the future to further validate the findings of this study. Furthermore, there is currently limited histopathological evidence to specifically validate NODDI metrics in human cortical grey matter. However, NODDI‐derived indices have been shown to closely correlate with histological counterparts on postmortem examination in the context of multiple sclerosis spinal cord lesions (Grussu et al., 2017). In addition, some discrepancies between the NODDI model and emerging multi‐shell diffusion techniques that utilize linear and spherical tensor encoding (as opposed to the single diffusion encoding approach of NODDI) have been reported (Lampinen et al., 2017). Refinement of multi‐shell diffusion analysis techniques is an important avenue of research and may enable further insights into grey matter microstructure in AD in the future.

Compared to the other ROIs, there was weaker evidence for significant AD‐related differences in NODDI metrics in the entorhinal cortex. This may reflect the fact that precise localization of this comparatively small region is difficult and combined with relatively large voxels used in diffusion MRI make it more prone to noise. A further limitation is the study's cross‐sectional nature and longitudinal datasets comprising participants at different stages of the AD pathological continuum (including presymptomatic cohorts) will be required to further tease out the temporal progression of cortical microstructural change in AD. Another focus for future research, as previously highlighted, would be to investigate associations between cortical microstructure and neuroimaging modalities that quantify other pathological processes in AD such as: beta‐amyloid (Clark et al., 2011; Joshi et al., 2012; Klunk et al., 2004) and tau deposition (Marquié et al., 2015), as well as neuroinflammation (Fan, Brooks, Okello, & Edison, 2017) and cerebrovascular disease (Sudre, Cardoso, & Ourselin, 2017) to further disentangle the key pathological drivers of alterations in cortical microstructure.

## 5 CONCLUSIONS

These data provide in vivo evidence of differences in the NODDI density and dispersion indices of neurites in the cortex of YOAD patients compared to age‐match controls, which persisted in the majority of ROIs following adjustment for cortical thickness. Differences in NDI were present in brain regions known to undergo early atrophy in AD, but also in a region typically spared of significant atrophy (precentral gyrus). Furthermore, there was also evidence that cortical NDI was associated with MMSE performance. These data suggest cortical NODDI metrics provide information regarding AD‐related pathology above and beyond that of cortical thinning alone and support the potential utility of cortical NODDI metrics as sensitive biomarkers of cortical pathology in AD.

## DECLARATION OF INTEREST

Catherine F. Slattery reports having received personal fees from GE Healthcare. Nick C. Fox reports personal fees from Janssen/Pfizer, IXICO, Roche, Lilly Research Laboratories (Avid), Novartis Pharma AG, Sanofi, and GSK (all fees paid to University College London). Jonathan M. Schott has received research funding from AVID Radiopharmaceuticals (a wholly owned subsidiary of Eli Lilly), has consulted for Roche, Eli Lilly, Biogen, and MSD, received royalties from Oxford University Press, given education lectures sponsored by Eli Lilly, and serves on a Data Safety Monitoring Committee for Axon Neuroscience SE. The remaining authors declared no conflicts of interest.

## Supporting information

Additional Supporting Information may be found online in the supporting information tab for this article.

Supporting Information
