# Supplementary material for: Cortical microstructure in young onset Alzheimer's disease using neurite orientation dispersion and density imaging
Source: Hum Brain Mapp. 2018 Mar 25;39(7):3005–17. doi: 10.1002/hbm.24056 (PMC6055830; doi:10.1002/hbm.24056)
Supplement: Supplementary file 1 — Supporting Information [file HBM-39-3005-s001.docx]

|  | | Typical YOAD (n=27) | Atypical YOAD (n=11) | p-value |
| --- | --- | --- | --- | --- |
| Age (years) | | 61.2 (5.1) | 61.0 (4.98) | 0.93^a^ |
| % Female | | 55.6 % | 72.3% | 0.47^b^ |
| Age at onset (years) | | 56.2 (4.4) | 56.6 (4.5) | 0.82^a^ |
| Disease Duration | | 4.9 (2.8) | 4.5 (2.2) | 0.59^a^ |
| MMSE score | | 20.7 (4.4) | 22.4 (5.2) | 0.28^c^ |
| Years of education | | 15.4 (2.5) | 15.7 (2.2) | 0.68^a^ |
| Entorhinal | Cortical thickness (mm) | 2.90 (0.38) | 2.98 (0.29) | 0.49^a^ |
|  | NDI | 0.261 (0.071) | 0.282 (0.087) | 0.5^a^ |
|  | ODI | 0.488 (0.028) | 0.495 (0.051) | 0.64^a^ |
| Inferior Temporal | Cortical thickness (mm) | 2.56 (0.2) | 2.52 (0.56) | 0.56^a^ |
|  | NDI | 0.295 (0.067) | 0.295 (0.051) | 0.99^a^ |
|  | ODI | 0.465 (0.035) | 0.462 (0.038) | 0.78^a^ |
| Middle Temporal | Cortical thickness (mm) | 2.53 (0.22) | 2.6 (0.2) | 0.38^a^ |
|  | NDI | 0.271 (0.068) | 0.279 (0.06) | 0.73^a^ |
|  | ODI | 0.511 (0.02) | 0.513 (0.022) | 0.75^a^ |
| Fusiform | Cortical thickness (mm) | 2.49 (0.21) | 2.24 (0.22) | 0.0051^**a^ |
|  | NDI | 0.319 (0.058) | 0.284 (0.046) | 0.059^a^ |
|  | ODI | 0.481 (0.03) | 0.466 (0.025) | 0.13^a^ |
| Precuneus | Cortical thickness (mm) | 2.00 (0.16) | 2.03 (0.22) | 0.69^a^ |
|  | NDI | 0.248 (0.07) | 0.249 (0.066) | 0.98^a^ |
|  | ODI | 0.485 (0.031) | 0.477 (0.03) | 0.39^a^ |
| Precentral | Cortical thickness (mm) | 2.40 (0.18) | 2.39 (0.22) | 0.89^a^ |
|  | NDI | 0.278 (0.063) | 0.270 (0.069) | 0.72^a^ |
|  | ODI | 0.481 (0.025) | 0.492 (0.028) | 0.73^a^ |

Supplementary Material. Comparison of typical (amnestic led presentation) and atypical (visual symptom-led posterior cortical atrophy phenotype). All data are mean (SD) unless stated otherwise

Key: YOAD = Young Onset Alzheimer’s Disease, n = number, SD = standard deviation, MMSE = Mini-Mental State Examination, n = number, NDI = neurite density index, ODI = Orientation Dispersion Index. ^a^Two-tailed t-test ^b^Two-sided Fisher’s exact ^c^Wilcoxon rank sum *p<0.05 **p<0.008 Bonferroni corrected threshold – p=0.05 divided by 6 (total number of regions of interest)
